# Edge-to-Edge Transcatheter Mitral Valve Repair Using PASCAL vs. MitraClip: A Systematic Review and Meta-Analysis

**DOI:** 10.3390/jcm12103579

**Published:** 2023-05-20

**Authors:** Kaveh Hosseini, Hamidreza Soleimani, Amir Nasrollahizadeh, Yaser Jenab, Angelos Karlas, Dimitrios V. Avgerinos, Alexandros Briasoulis, Toshiki Kuno, Ilias Doulamis, Polydoros N. Kampaktsis

**Affiliations:** 1 Cardiac Primary Prevention Research Center, Cardiovascular Diseases Research Institute, Tehran University of Medical Sciences, Tehran 1419733141, Iran; kaveh_hosseini130@yahoo.com (K.H.); hamid.r.soleimani90@gmail.com (H.S.); nasrollahi.amir@yahoo.com (A.N.); yasjenab@gmail.com (Y.J.); 2 Tehran Heart Center, Cardiovascular Diseases Research Institute, Tehran University of Medical Sciences, Tehran 1419733141, Iran; 3 Non-Communicable Diseases Research Center, Endocrinology and Metabolism Population Sciences Institute, Tehran University of Medical Sciences, Tehran 1411713139, Iran; 4 Institute for Biological and Medical Imaging, Helmholtz Zentrum München, 85764 Neuherberg, Germany; angelos.karlas@tum.de; 5 Department for Vascular and Endovascular Surgery, Rechts der Isar Hospital, Technical University of Munich, 81675 Munich, Germany; 6 Onassis Cardiac Surgery Center, 17674 Athens, Greece; davgerinos@gmail.com; 7 Medical School, National and Kapodistrian University of Athens, 11527 Athens, Greece; alexbriasoulis@gmail.com; 8 Department of Medicine, Montefiore Medical Center, New York, NY 10461, USA; tkuno@montefiore.org; 9 Department of Surgery, The Johns Hopkins Hospital, School of Medicine, Baltimore, MD 21287, USA; doulamis.i@gmail.com; 10 Division of Cardiology, Columbia University Irving Medical Center, New York, NY 10032, USA

**Keywords:** transcatheter edge-to-edge repair, mitral regurgitation, MitraClip, PASCAL

## Abstract

Background: Transcatheter edge-to-edge repair (TEER) of the mitral valve (MV) can be performed using the PASCAL or MitraClip devices. Few studies offer a head-to-head outcome comparison of these two devices. Material and Methods: PubMed, EMBASE, Cochrane Library, Clinicaltrials.gov and WHO’s International Clinical Trials Registry Platform, from 1 January 2000 until 1 March 2023, were searched. Study protocol details were registered in the International Prospective Register of Systematic Reviews (PROSPERO ID: CRD42023405400). Randomized Controlled Trials and observational studies reporting head-to-head clinical comparison of PASCAL and MitraClip devices were eligible for selection. Patients with severe functional or degenerative mitral regurgitation (MR) who had undergone TEER of the MV with either PASCAL or MitraClip devices were included in the meta-analysis. Data from six studies (five observational and one randomized clinical trial) were extracted and analyzed. The main outcomes were a reduction in MR to 2+ or less, improvement of New York Heart Association (NYHA) and 30-day all-cause mortality. Peri-procedural mortality, success rate and adverse events were also compared. Results: Data from 785 and 796 patients that underwent TEER using PASCAL and MitraClip, respectively, were analyzed. Thirty-day all-cause mortality (Risk ratio [RR] = 1.51, 95% CI 0.79–2.89), MR reduction to maximum 2+ (RR = 1.00, 95% CI 0.98–1.02) and NYHA improvement (RR = 0.98, 95% CI 0.84–1.15) were similar in both device groups. Both devices had high and similar success rates (96.9% and 96.7% for the PASCAL and MitraClip group, respectively, *p* value = 0.91). MR reduction to 1+ or less at discharge was similar in both device groups (RR = 1.06, 95% CI 0.95–1.19). Composite peri-procedural and in-hospital mortality was 0.64% and 1.66% in the PASCAL and MitraClip groups, respectively (*p* value = 0.094). Rates of peri-procedural cerebrovascular accidents were 0.26% in PASCAL and 1.01% in MitraClip (*p* value = 0.108). Conclusions: Both PASCAL and MitraClip devices have high success and low complication rates for TEER of the MV. PASCAL was not inferior to MitraClip in reducing the MR level at discharge.

## 1. Introduction

The paradigm of surgical approaches for valvular disease has been challenged by minimally invasive, catheter-based therapies [1]. Development of transcatheter edge-to-edge repair (TEER) techniques for the treatment of mitral regurgitation (MR) has received interest, especially in patients with high surgical risk (defined as patients with a Society of Thoracic Surgeons-predicted risk of mortality > 8% (STS-PROM)) [2,3]. Compared to medical treatment of patients with moderate-to-severe or severe secondary MR, TEER has been shown to result in a lower risk for hospitalization for heart failure or all-cause mortality [4].

MitraClip (Abbott Vascular, Santa Clara, CA, USA) and PASCAL (Edwards Lifesciences, Irvine, CA, USA) mitral valve repair systems have been developed and are currently FDA-approved for TEER treatment of MR in specific populations. As the first FDA-approved transcatheter repair system and using a mechanism based on the Alfieri suture, MitraClip has been shown to reduce MR severity in high-risk surgical patients [5]. Unlike earlier generation MitraClip devices, PASCAL allows for independent leaflet capture and has a Nitinol spacer between the clasping arms, easing the strain on leaflets. It has also been suggested that the PASCAL device provides a more user-friendly steering mechanism [6].

Available data supporting the safety and efficacy of MitraClip exceed those of PASCAL, and studies offering a head-to-head outcome comparison of these two techniques are limited. To the best of our knowledge, there has not yet been a systematic review and meta-analysis comparing these two systems in terms of patient selection, immediate and mid-term success rate, incidence of adverse events and mortality.

## 2. Methods

### 2.1. Design and Search Strategy

This systematic review and meta-analysis followed the Preferred Reporting Items for Systematic Reviews and Meta-Analysis (PRISMA) guidelines [7]. Study protocol details were registered in the International Prospective Register of Systematic Reviews (PROSPERO) [8]. (PROSPERO ID: CRD42023405400) [9].

After identifying relevant keywords and search terms (Supplementary Materials), we performed a systematic search in the following electronic databases: PubMed, EMBASE, Cochrane Library, Clinicaltrials.gov and WHO’s International Clinical Trials Registry Platform, from 1 January 2000 until 1 March 2023. No language restriction was applied. A reference list of eligible studies and relevant reviews was also screened. 

### 2.2. Selection Criteria

Randomized Controlled Trials (RCTs) and observational studies reporting head-to-head clinical comparison of PASCAL and MitraClip devices were eligible for selection. Non-comparative studies and cohort studies that reported the outcomes only on one of the devices were excluded. Abstracts, case reports, review articles, trial design protocols, non-comparative studies and conference abstracts were dismissed. 

Patients with severe functional or degenerative MR who had undergone TEER with PASCAL or MitraClip devices were eligible for inclusion in this study. 

### 2.3. Outcomes

The primary outcomes were the following: rate of MR reduction to 2+ or less at the time of discharge, short-term mortality defined as all-cause mortality in the first 30 days or up until the first follow-up visit after completion of the procedure and improvement of New York Heart Association (NYHA) functional class to class II or less at 30 days post-TEER. Incidence of adverse events, procedural success rates and rates of MR reduction to 1+ or less at the time of discharge were also compared. Definition of safety outcomes was based on the Mitral Valve Academic Research Consortium (MVARC) Criteria [10]. Data on baseline demographic, clinical and echocardiographic characteristics of patients and incidence of procedural adverse events were also summarized.

### 2.4. Data Collection and Management

Results of the systematic search were imported into Endnote software version 20.0 (Clarivate PLC, London, UK). Title and abstract of each entry were screened by two independently working reviewers (HS, AN), and a third reviewer (KH) resolved any arising conflicts. After retrieving the full text of selected studies, data were extracted using a predesigned form. Data regarding the name of the first author, study site, study type, sample size of each of the comparison groups, baseline characteristics, incidence of adverse events and primary and secondary outcomes were collected in this step. 

### 2.5. Risk of Bias Assessment

Version 2.0 of Cochrane Risk of Bias Assessment Tool for Randomized Trials (RoB2) [11,12] was used to assess the quality of RCTs. The Newcastle–Ottawa Scale (NOS) [13] was used to assess the quality of non-randomized studies. Selection, comparability and ascertainment of exposure/outcome were assessed in each non-randomized study; two authors assigned stars in each of the categories and conflicts were resolved by way of consensus. 

### 2.6. Data Analysis and Investigation of Heterogeneity

All statistical analyses were conducted with R programming language (R for Windows, version 4.1.3, Vienna, Austria), R Studio version 1.1.463 (Posit PBC, Boston, MA, USA) utilizing the “tidyverse” and “meta” statistical packages. For binary variables, risk ratios with 95% confidence intervals were calculated. For continuous variables, mean and standard deviation (SD) were calculated; in studies that reported median and interquartile (IQR) ranges, we used the method developed by Lou et al. [14] and Wan et al. [15] to calculate mean and SD. Heterogeneity was assessed using the *I*^2^ statistic; significant heterogeneity was defined as *I*^2^ > 70%. We used a random effects model to estimate the effect size of the pooled data. Funnel plots were not produced for this study as the total number of studies included was lower than 10. 

## 3. Results

### 3.1. Study Selection

**Figure 1:**
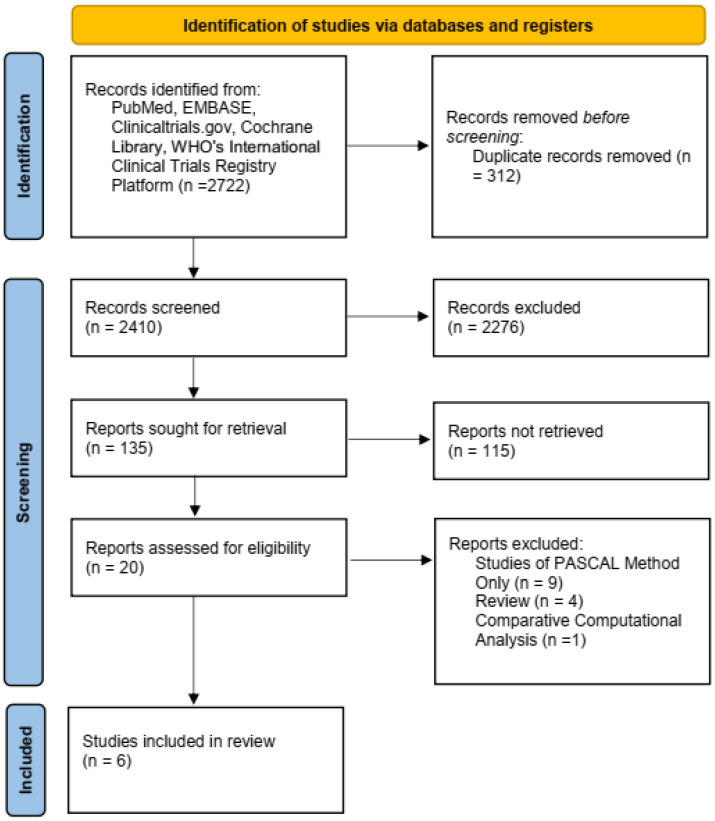
Schematic overview of study selection.

Our search yielded a total of 2722 references, 2410 of which remained after eliminating duplicates. After the first step of screening and retrieval of qualified studies, 20 studies were assessed for eligibility. Finally, we analyzed data from six studies (five cohorts and one RCT) published between 2021 and 2022 (Figure 1).

### 3.2. Study Characteristics

Selected studies are summarized in Table 1. Five studies were observational analyses, and one was RCT [16], namely the CLASP IID trial (Edwards PASCAL Transcatheter Valve Repair System Pivotal Clinical Trial, NCT03706833 [17]). All of the observational studies were conducted in Germany, and all study results were published between 2021 and 2022.

### 3.3. Quality Assessment

The risk of bias of the RCT included in the analysis was estimated to be low, and a reasonable randomization procedure was used for enrolling patients in each arm (Table 2). The results of the quality assessment are explained extensively in Supplementary Materials.

### 3.4. Baseline Characteristics

This meta-analysis pooled data from 785 patients that underwent TEER using the PASCAL device and 796 that underwent TEER using the MitraClip device. Mauri et al. and Schneider et al. reported a statistically significant difference between PASCAL and MitraClip groups regarding the European System for Cardiac Operative Risk Evaluation Score (EUROScore) (6.9 ± 4.6% for MitraClip vs. 5.8 ± 4.5% for PASCAL, *p* value = 0.002 [21] and (7.2 ± 7.0% for MitraClip vs. 5.8 ± 4.9% for PASCAL, *p* value = 0.06, respectively [22]). The same two studies reported a difference in left ventricular end-systolic diameter (LVESD) (46 ± 12 mm for MitraClip vs. 44 ± 13 mm for PASCAL, *p* value = 0.044 [21] and 45 ± 13 mm for MitraClip vs. 42 ± 13 mm for PASCAL, *p* value = 0.009, respectively [22]). All other included studies reported no difference between the groups in terms of baseline characteristics, including EURO Score, N-terminal prohormone brain natriuretic peptide levels, pulmonary artery systolic pressure, left ventricular ejection fraction, LVESD, left ventricular end-diastolic diameter and NYHA functional capacity class (Table 3). When data were pooled together, there was no significant difference between groups regarding the mechanism of MR (functional/mixed vs. degenerative). Only one study reported a significant difference between the number of implanted devices: TEER required more than one device less frequently when the PASCAL vs. MitraClip systems were used (24.1% vs. 39.4%, *p* value < 0.001) [21]. Lim et al. showed that TEER with PASCAL required on average 9 min longer compared to MitraClip TEER (88 min, IQR = 68.5–122 min vs. 79 min, IQR = 58–106 min, *p* value = 0.023) [16]. All included studies reported the mean transmitral gradient (MG) at the time of discharge. In the study by Mauri et al., MG was significantly higher in the MitraClip group (3.9 ± 1.7 mmHg vs. 3.3 ± 1.mmHg, *p* value < 0.001) [21]. In the study by Schneider et al., MG was similar in both groups (3.6 ± 1.6 mmHg vs. 3.4 ± 1.6 mmHg, *p* Value = 0.16 [22]). Similarly, no difference was seen in the study by Haschemi et al. (3 mmHg, IQR = 2–4 mmHg for MitraClip vs. 3 mmHg, IQR = 2–4 mmHg for PASCAL, *p* value = 0.519) [20].

## 4. Outcomes

### 4.1. Primary Outcomes

**Figure 2:**
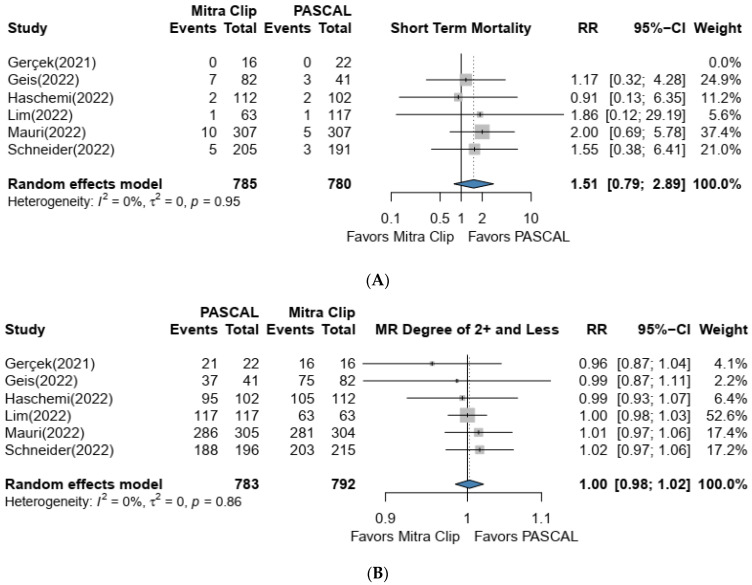

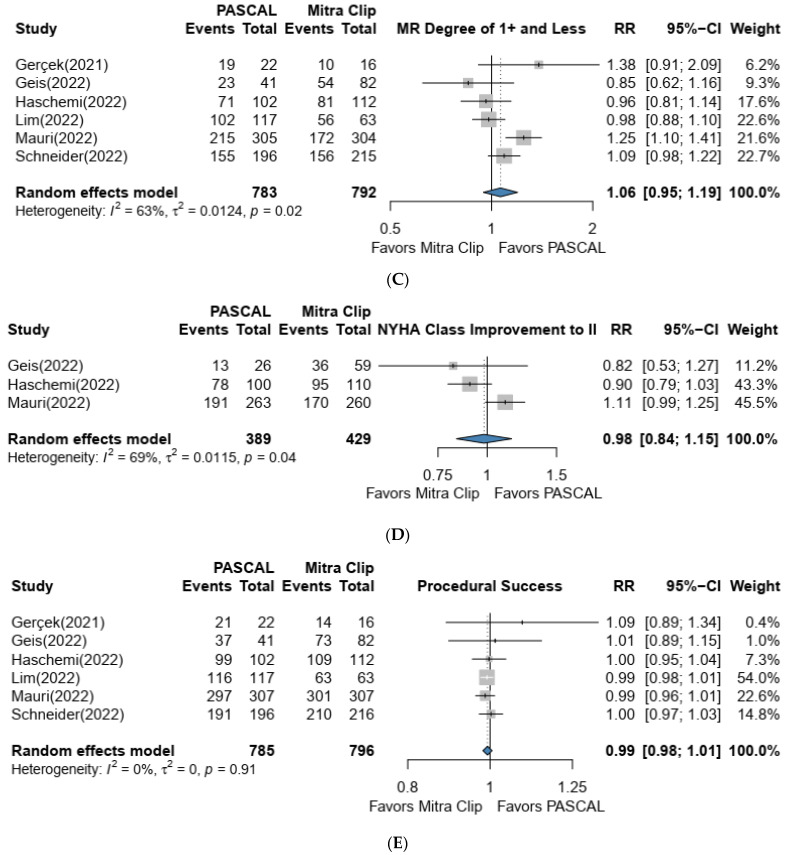
(**A**–**E**) Forest plot showing the observed outcomes and the estimate of the random effects model for Short-term mortality, MR ≤ 2+ at Discharge, MR ≤ 1+ at Discharge, NYHA class ≤ 2 at first Follow up and Success rate [16,18,19,20,21,22].

There was no significant difference in short-term all-cause mortality rates when comparing PASCAL vs. MitraClip groups (RR: 1.52, 95% CI 0.80–2.90, *p* value = 0.95), with low heterogeneity (*I*^2^ = 0%) (Figure 2A). Results of the pooled analysis revealed no statistically significant difference in the rates of MR reduction to 2+ or less at the time of discharge [RR: 1.01, 95% CI 0.98–1.03, *p* value = 0.86), with low heterogeneity (*I*^2^ = 0%) (Figure 2B). Only three studies reported NYHA class at the first follow-up assessment. Pooled analysis via fixed effect model demonstrated no significant difference between the two devices (RR: 1.02, 95% CI 0.94–1.12, *I*^2^ = 69%); (Figure 2D).

### 4.2. Secondary Outcomes

In all six studies, both systems had high success rates (96.9% and 96.7% for the PASCAL and MitraClip groups, respectively), with no difference between the two systems (RR: 1.0, 95% CI 0.98–1.02, *p* = 0.86, *I*^2^ = 0%) (Figure 2E). By investigating the data from included studies, we found that the PASCAL system was not inferior to MitraClip in reducing regurgitation to MR ≤ 1+ at the time of discharge [RR: 1.06, 95% CI 0.95–1.19, *p* value = 0.02). However, heterogeneity was high (*I*^2^ = 63%) (Figure 2C).

### 4.3. Adverse Events

Procedural-related mortality, cerebrovascular accident (CVA), major bleeding and need for reintervention were reported in all six included studies. In general, incidences of complications were low. Table 4 summarizes adverse events. To assess mortality as an adverse event, we used a composite of procedural and post-procedural in-hospital mortality (all deaths that occurred before hospital discharge). In most of the included studies, MitraClip had a higher procedural-related mortality rate compared to PASCAL; composite mortality was 0.64% and 1.66% in the PASCAL and MitraClip group, respectively (*p* value = 0.094). The pooled incidence of CVA in MitraClip was four times that of the PASCAL. However, this difference was not statistically significant (0.26% vs. 1.01% for PASCAL vs. MitraClip, respectively, *p* value = 0.108). Data from six studies were aggregated for meta-analysis, and subsequently, no significant distinction was shown regarding major bleeding (PASCAL vs. MitraClip bleeding incidence was 1.79% vs. 1.01%, *p* value = 0.205). Five of the six final included studies reported the rate of reintervention (except Haschemi et al.) [20] as the most frequent adverse event observed (per study numbers). Extracted data underwent pooled analysis, and the incidence of reintervention was 1.03% and 1.34% in the PASCAL and MitraClip group, respectively, *p* value = 0.925).

## 5. Discussion

Clinical trials for both MitraClip and PASCAL systems have yielded good safety, efficacy and improved outcomes for certain populations [21,23]. Both the PASCAL system and MitraClip device are utilized for treating MR, but their level of efficacy evidence differs. The MitraClip device has undergone the CoAPT trial [23], which revealed its effectiveness in reducing mortality and hospitalizations, whereas the PASCAL system has yet to produce data from RCTs. Therefore, the MitraClip device has stronger supporting evidence than the PASCAL system. Additional research is necessary to gain a better understanding of the PASCAL system’s effectiveness when compared to other devices and to establish its level of evidence. However, there are certain distinctions between two devices that might make one strategy more appropriate for particular individuals depending on factors such as anatomical features [24]. So far, studies comparing the two devices have had small sample sizes. To the best of our knowledge, this is the first systematic review to compare the outcomes and adverse events of PASCAL and MitraClip systems. The main finding of our meta-analysis is that MitraClip and PASCAL systems are both effective for TEER of MR without differences in safety outcomes. The PASCAL device may be more effective in reducing MR to 1+ or less.

### 5.1. Outcomes

Short-term mortality was low and without a significant difference when PASCAL or MitraClip devices were used. There are two points to consider here: first, most of the studies have not offered intermediate and long-term mortality rates, thus limiting the comparison only to short-term outcomes. Second, most studies did not distinguish between cardiovascular and non-cardiovascular deaths. Further studies with larger sample sizes, longer follow-up time and the distinction between cardiovascular and non-cardiovascular death are required to thoroughly compare the two devices.

Regarding the efficacy of the two devices, MR reduction to a maximum of 2+ was not different between groups.

Despite the favorable results of two studies with large sample sizes (Gercek and Mauri) in favor of the PASCAL system for reducing MR to a maximum of 1+ [18,21], our combined analysis showed no difference between the groups. The results suggest that the PASCAL system is not inferior in reducing MR to 1+ or less, but due to high heterogeneity, further RCTs are needed to clarify this issue. An interesting area for investigation is the comparison of future outcomes between groups with 1+ or 2+ MR at discharge, including the need for reintervention and recurrence of symptoms. Currently, it is unclear whether better MR reduction results will lead to improved clinical outcomes in the future.

Improvement of NYHA class was the least reported outcome in our meta-analysis. In contrast to the Geis and Haschemi studies [19,20], the Mauri study [21] had the biggest sample size and reported better improvement in NYHA class for the PASCAL group. Overall, the lack of difference between the two groups may be due to small sample sizes. Heterogeneity in reporting and gathering data across studies, as well as the absence of unified measurement criteria and standard pre-defined follow-up time periods, restricted our ability to perform a more thorough analysis. NYHA functional class is a crucial and simple to evaluate functional index, and its utilization is critical in studies comparing TEER strategies.

The success rate of TEER procedures has been demonstrated to be both high and without differences [25]. Furthermore, in the Lim study [16], the only randomized clinical trial comparing PASCAL and MitraClip for which the results were available at the time of preparing this paper, the success rate in both groups was reported to be greater than 99%.

### 5.2. Adverse Events

TEER has helped to reduce complications associated with open heart surgery [26]. Overall, there were only 4 and 12 instances of procedural-related mortality in the PASCAL and MitraClip groups, respectively, with no statistically significant difference.

Regarding CVA, the incidence was four times higher in the MitraClip group. While the final calculated effect size was not significant, it is worth noting that in two of the studies [19,21], there was a considerable difference in terms of CVA incidence; in both studies, there were more CVAs recorded in the MitraClip group. However, this difference was not significant, mainly due to the very low total incidence of CVA. Additionally, our findings may be influenced by the fact that the studies we analyzed did not provide a clear definition or specific paraclinical test for diagnosing CVA or stroke.

Reintervention following TEER is considered to be an independent risk factor for mortality [27] and, therefore, an important complication. Based on our findings incidence of reintervention was low and comparable between the two systems (1.33% and 1.48% in PASCAL and MitraClip groups, respectively).

### 5.3. Limitations

There are some limitations to our study. First, there has only been one RCT conducted so far, the completed results of which are not published yet, and there may be notable limitations in terms of selection and reporting biases, ultimately affecting the overall quality of our pooled analysis. Secondly, our findings could be biased due to the small sample sizes of included studies. This particularly holds true for adverse events, which have a low incidence. Third, some echocardiographic features could identify the system of choice for individualized MR repair, but relevant data are limited, and we need more studies with more uniform reporting standards to further clarify the issue. Fourth, different studies use different definitions, measurement criteria and timeframes for reporting their safety and efficacy outcomes, which hampered our ability to provide uniform analysis. Particularly, we had to use all-cause mortality, which reduces the sensitivity of the eventual estimate. Finally, given that each of the included studies had different follow-up time frames and most of the studies only reported results from short-term follow-ups, intermediate and long-term comparison of the outcomes and adverse events of PASCAL and MitraClip groups was not possible.

## 6. Conclusions

The PASCAL TEER system appears to have similar efficacy and safety compared to the MitraClip for the treatment of MR. The PASCAL system showed similar rates of MR reduction at discharge. Results from future RCTs will shed more light on the topic, particularly in regard to whether each system is better for specific patients.

## Figures and Tables

**Table 1:** Characteristic and demographic information of included studies.

First Author and Year	Study Region	Study Type	Number of Patients		Age (Mean ± SD)		Male Sex %		Outcome
			PASCAL	MitraClip	PASCAL	MitraClip	PASCAL	MitraClip	
Gercek 2021 [18]	Germany	Retrospective	22	16	81.9 ± 6.2	81.8 ± 8.1	59.1%	56.2%	Short-term follow-up period was within 30 days after implantation. Reduction of MR to grade ≤ 1+ was significantly more frequent in PASCAL group. Safety and other outcomes were similar in both TEER systems.
Geis 2022 [19]	Germany	Retrospective	41	82	74.4 ± 13.9	77.5 ± 14.2	58.5%	54.9%	A short-term follow-up time frame was between 30 days and 4 months (first visit). Although aborted implantation due to elevated MPG was seen more in PASCAL group, technical success was similar in both systems. Additionally, short-term and 1-year outcomes were noninferior in PASCAL group compared to MitraClip group.
Haschemi 2022 [20]	Germany	Prospective	102	112	NA	NA	NA	NA	Short-term follow-up was 30 days after TEER. No significant difference in technical success, mean valvular gradient, MR degree of ≤2+ at 1st month and discharge and mortality was seen between two groups.
Lim 2022 [16]	United States, Canada and Europe	RCT (Interim Analysis)	117	63	81.1 ± 6.9	81.2 ± 6.2	66.7%	68.3%	Short-term follow-up period was within 30 days after implantation. PASCAL and MitraClip groups showed similar incidences of major adverse events in first month and MR reduction to ≤2+ at six months
Mauri 2022 [21]	Germany	Retrospective	307	307	77 ± 9.6	77.1 ± 8.5	57.7%	58.0%	Short-term follow-up visits were scheduled for 30 days. Technical success, procedure time, major adverse events and degree of MR ≤ 2+ at discharge were comparable in both groups, but higher rate of MR reduction to grade ≤ 1+ and a transmitral pressure gradient below 5 mm Hg was achieved in PASCAL group
Schneider 2022 [22]	Germany	Retrospective	196	216	76 ± 12	77 ± 9	61.2%	50.5%	Short-term follow-up period was within 30 days after implantation. Residual MR ≤ 1+, technical success rates, 30-day mortality and long-term outcomes were similar in both groups

SD, standard deviation; NA, not applicable; RCT, randomized control trial; MR, mitral regurgitation; TEER, transcatheter edge-to-edge repair; MPG, mean pressure gradient.

**Table 2:** Quality assessment of cohort studies.

First Author and Year	Selection	Comparability	Exposure/Outcome	Total Score
Gercek 2021 [18]	****	**	***	*********
Geis 2022 [19]	****	**	***	*********
Haschemi 2022 [20]	****	**	***	*********
Mauri 2022 [21]	****	*	***	********
Schneider 2022 [22]	****	*	***	********

Good quality: 3 or 4 stars in selection domain AND 1 or 2 stars in comparability domain AND 2 or 3 stars in outcome/exposure domain. Fair quality: 2 stars in selection domain AND 1 or 2 stars in comparability domain AND 2 or 3 stars in outcome/exposure domain. Poor quality: 0 or 1 star in selection domain OR 0 stars in comparability domain OR 0 or 1 stars in outcome/exposure domain.

**Table 3:** Patient baseline characteristics.

First Author and Year	EURO Score II (Mean ± SD)		NT-Pro-BNP (Mean ± SD)		SPAP (Mean ± SD)		LVEF (Mean ± SD)		LVESD (Mean ± SD)		LVEDD (Mean ± SD)		NYHA Class ≥ 3	
	P	M	P	M	P	M	P	M	P	M	P	M	P	M
Gercek 2021 [18]	4.7 ± 3.7	4.3 ± 3	2941 ± 3271	3032 ± 2696	43 ± 20.7	53.4 ± 21.3	NA	NA	NA	NA	NA	NA	100%	100%
Geis 2022 [19]	5.1 ± 3.7	6.6 ± 7.4	4519 ± 7050	5575 ± 6993	53 ± 13.8	49 ± 13.6	40.1 ± 29.2	40 ± 21.1	44.3 ± 16.9	43.5 ± 13.6	55 ± 12.3	56 ± 10.5	87.8%	87.8%
Haschemi 2022 [20]	NA	NA	NA	NA	NA	NA	NA	NA	NA	NA	NA	NA	80.4%	78.6%
Lim 2022 [16]	3.9 ± 2.9	4.1 ± 3.1	NA	NA	42.3 ± 11.4	45.6 ± 14.6	59.6 ± 8.7	58.3 ± 9	38.3 ± 7.7	39.8 ± 7.8	57.1 ± 6.5	57.4 ± 6.5	60.7%	61.9%
Mauri 2022 [21]	5.8 ± 4.5	6.9 ± 4.9	NA	NA	45 ± 14	49 ± 16	47 ± 15	47 ± 15	44 ± 13	46 ± 12	57 ± 10	57 ± 10	86.0%	83.1%
Schneider 2022 [22]	5.8 ± 4.9	7.2 ± 7	5084 ± 7197	5825 ± 8298	44 ± 16	52 ± 16	50 ± 15	47 ± 15	42 ± 13	45 ± 13	57 ± 11	57 ± 11	91.3%	85.2%
Total	5.4 ± 4.4	6.6 ± 5.6	4834.5 ± 6994.9	5676.3 ± 7894.9	44.7 ± 14.5	49.8 ± 15.8	49.7 ± 15.4	47.2 ± 15.5	42.4 ± 12.5	44.8 ± 12.2	50.9 ± 11.5	51.9 ± 11.1	83.3%	82.2%

SD, standadrd deviation; NA, not applicable; P, PASCAL; M, MitraClip; EURO Score, European System for Cardiac Operative Risk Evaluation; NT-pro-BNP, N-terminal prohormone brain natriuretic peptide; SPAP, systolic pulmonary arterial pressure; LVEF, left ventricular ejection fraction; LVESD, left ventricular end-systolic diameter; LVEDD, left ventricular end-diastolic diameter; NYHA, New York Heart Association. Data for Haschemi study was expressed in median and interquartile ranges; as the data was skewed, it was not possible to calculate mean and SD for results and show them in the table.

**Table 4:** Adverse events.

First Author and Year	Mortality		CVA		Bleeding		Reintervention	
	PASCAL	MitraClip	PASCAL	MitraClip	PASCAL	MitraClip	PASCAL	MitraClip
Gercek 2021 [19]	0	0	0	0	0	0	0	0
Geis 2022 [18]	0	5	0	2	0	2	0	3
Haschemi 2022 [20]	1	1	1	0	0	0	NA	NA
Lim 2022 [16]	0	1	0	0	3	2	1	0
Mauri 2022 [21]	1	1	1	5	7	3	3	3
Schneider 2022 [22]	3	5	0	1	4	1	4	2
Total (percent)	5 (0.64%)	13 (1.66%)	2 (0.26%)	8 (1.01%)	14 (1.79%)	8 (1.01%)	8 (1.33%)	8 (1.19%)
p value	0.094		0.108		0.205		0.925	

NA, not applicable.

## Data Availability

Search strategy file was uploaded as a supplementary file.

## Supplementary Materials

The following supporting information can be downloaded at: https://www.mdpi.com/article/10.3390/jcm12103579/s1.
