# Supplementary material for: Edge-to-Edge Transcatheter Mitral Valve Repair Using PASCAL vs. MitraClip: A Systematic Review and Meta-Analysis
Source: J Clin Med. 2023 May 20;12(10):3579. doi: 10.3390/jcm12103579 (PMC10219028; doi:10.3390/jcm12103579)
Supplement: Supplementary file 1 [file jcm-12-03579-s001.zip › jcm-2381972-supplementary.pdf]

PUBMED 1772 RESULTS

((((((((((("Mitral Valve Insufficiency"[Mesh] AND ("PASCAL" OR "edge-to- edge technique" OR "percutaneous" OR "transcutaneous" OR "transcatheter" OR "catheter-based" OR "endo-vascular" OR "trans-septal" OR "Transcatheter edge-to-edge repair" OR "TEER" OR "mitra clip" OR "mitraclip")) NOT ("tavi")) NOT ("aorta")) NOT ("paravalvular")) NOT ("leak")) NOT ("leakage")) NOT ("echo")) NOT ("echocardiography")) NOT ("transapical")) NOT ("tavr")) NOT ("amplatz")) NOT ("atrial septal defect")) NOT ("valve in valve") AND (2000/1/1:2023/3/1[pdat])

EMBASE 949 RESULTS

('edge to edge mitral valve repair'/exp OR 'mitral valve clip'/exp OR 'valvuloplasty catheter'/exp) AND 'mitral valve regurgitation'/exp NOT ('transcatheter aortic valve implantation' OR 'aortic disease' OR 'echocardiography' OR 'transthoracic echocardiography' OR 'transesophageal echocardiography' OR 'heart atrium septum defect' OR 'interatrial septum' OR 'paravalvular leak' OR 'paravalvular leakage' OR 'paravalvular regurgitation' OR 'paravalvular leak device' OR 'valve-in-valve transcatheter aortic valve implantation') AND [2000-2023]/py

2721 RESULTS

312 DUPLICATES

2409 FINAL RESULTS

After title screening => 134
